# Rapid On-Site Detection of Zearalenone in Maize Using a Colloidal Gold Immunochromatographic Strip

**DOI:** 10.3390/bios15120810

**Published:** 2025-12-12

**Authors:** Mengjiao Wu, Xiaofei Hu, Lu Fan, Bo Wan, Yaning Sun, Yunrui Xing, Lianjun Song, Xianqing Huang, Mei Hu, Gaiping Zhang

**Affiliations:** 1 College of Food Science and Technology, Henan Agricultural University, Zhengzhou 450002, China; wumj826@163.com (M.W.);; 2 Longhu Laboratory of Advanced Immunology, Zhengzhou 450046, China; 3 Institute for Animal Health, Henan Academy of Agricultural Sciences (Key Lab for Animal Immunology, Henan Academy of Agricultural Sciences), Zhengzhou 450002, China; huxf1972@126.com (X.H.);

**Keywords:** colloidal gold immunochromatographic assay (CG-ICA), zearalenone (ZEN), food safety

## Abstract

Zearalenone (ZEN), a stable mycotoxin with estrogenic activity produced by various *Fusarium* species, poses a serious food safety risk. To facilitate the rapid, sensitive, on-site detection of ZEN in maize and ensure consumer dietary safety, a colloidal gold immunochromatographic assay (CG-ICA) based on a monoclonal antibody was established. ZEN was converted via oxime derivatization into hapten ZAN-O, which was conjugated to a carrier protein to prepare an immunogen for producing a highly specific and sensitive monoclonal antibody. Then, the antibody was conjugated into colloidal gold nanoparticles (AuNPs) and used as capture bioprobes of the CG-ICA test strip. The highly sensitive and specific detection platform was established through systematic optimization of pH value, coating antigen concentration, antibody-labeling dosage, incubation time, and strip assembly conditions. Under optimized conditions, the strip exhibited a detection limit of 11.79 pg/mL and an IC_50_ of 99.06 pg/mL, with a linear detection range of 13.40–732.48 pg/mL. In addition, the anti-interference capability assay demonstrated that the developed test strip possessed excellent specificity. In spiked maize samples, the CG-ICA test strip demonstrated recoveries ranging from 85.36% to 98.86%, with relative standard deviations (RSDs) below 10%. Thus, the CG-ICA strip provides a rapid, sensitive, and robust on-site tool for ZEN screening in maize, and can be adapted to other hazards by simply switching the antibody.

## 1. Introduction

Zearalenone (ZEN), also known as F-2 toxin, is a secondary metabolite produced by various *Fusarium* species, especially *Fusarium graminearum*, which predominantly contaminates cereals such as maize and wheat [1]. ZEN has a molecular weight of 318.36 g/mol. To generate specific antibodies, a ZEN-derived hapten containing an appropriate functional group was designed and conjugated with a carrier protein, while preserving the characteristic structural features of ZEN. Due to its exceptional structural stability and thermal resistance, conventional processing methods always fail to eliminate ZEN residue, resulting in its accumulation in contaminated grains, posing a significant food safety risk [2]. Since ZEN is structurally analogous to endogenous estrogens, it can bind to estrogen receptors and disrupt endocrine signaling pathways, thus resulting in reproductive [3,4] and immune issues and neurotoxicity [5]. Furthermore, ZEN can also trigger oxidative stress and apoptosis, which contribute to hepatic and immune injury [6,7,8,9]. In 2017, the International Agency for Research on Cancer (IARC) listed ZEN as a Group 3 (not classifiable regarding carcinogenicity to humans) agent [10]. To protect public health, strict maximum residue limits (MRLs) for ZEN in cereals have been implemented globally: in the USA, the MRLs are set in the range of 20–100 µg/kg [11]; in the EU, it is 350 µg/kg for raw maize and 100 µg/kg for feed [12]; in France, the limit is 50 µg/kg [13]; and in China, it is mandated at a maximum of 60 μg/kg in cereals and their products such as wheat, wheat flour, corn, and corn flour. Moreover, several international organizations have issued recommendations concerning dietary exposure to ZEA. For example, the Joint FAO/WHO Expert Committee on Food Additives (JECFA) established a provisional maximum tolerable daily intake (PMTDI) of 0.5 µg/kg body weight per day [14], whereas the European Food Safety Authority (EFSA) proposes a more conservative tolerable daily intake (TDI) of 0.25 µg/kg body weight per day [15]. Hence, it is essential to construct a quantitative method for the rapid detection and monitoring of ZEN.

Current analytical options for ZEN include thin-layer chromatography (TLC) [16], high-performance liquid chromatography (HPLC) [17], liquid chromatography–mass spectrometry (LC-MS), Raman spectroscopy [18], electrochemical detection [19], the enzyme-linked immunosorbent assay (ELISA) [20], and the colloidal gold immunochromatographic assay (CG-ICA) [21]. Although instrumental methods such as LC-MS offer high sensitivity, they require time-consuming sample clean-up and costly instrumentation, making them unsuitable for on-site screening [22]. CG-ICA strips are inexpensive, user-friendly, portable, and rapid, and have been widely adopted for field testing [23]. These immunoassays rely on antigen–antibody recognition and provide qualitative or semi-quantitative results within several minutes.

This study aims to develop a portable CG-ICA based on Au NPs for the rapid, accurate, and sensitive on-site detection of ZEN in maize and processed cereals. Au NPs exhibit ideal properties as signal bioprobes, such as a high molar extinction coefficient, exceptional chemical stability, and superior biocompatibility, making them particularly well-suited for highly sensitive bioassays. By conjugating ZEN monoclonal antibodies (mAbs), the Ab-AuNP bioprobes were prepared for constructing the CG-ICA test strips. Under optimal experimental conditions, the aggregation of bioprobes on the nitrocellulose (NC) membrane generated a colorimetric signal inversely proportional to ZEN concentration via an indirect competitive assay, allowing for sensitive quantification in maize extracts. The present work delivers essential technical support for food safety surveillance and facilitates the extended application of colloidal gold immunochromatography in small-molecule toxin analysis.

## 2. Materials and Methods

### 2.1. Animals, Reagents, and Equipment

Female BALB/c mice with good growth status of SPF grade were purchased from Henan Skobes Biotechnology Co., Ltd. (Anyang, China). Animal protocols were approved by the local ethics committee (approval No. LLSC41024017). Standard solutions of zearalenone (ZEN), α-zearalenol, β-zearalenol, aflatoxin B_1_, deoxynivalenol, T-2 toxin, and ochratoxin A were purchased from Tanmo Quality Inspection Standard Substances Center (Changzhou, China). N-Ethyl-N′-(3-dimethylaminopropyl) carbodiimide hydrochloride (EDC) and horseradish peroxidase (HRP)-conjugated goat anti-mouse IgG were obtained from Thermo Scientific (Waltham, MA, USA). Bovine serum albumin (BSA), ovalbumin (OVA), N-hydroxysuccinimide (NHS), Freund’s complete adjuvant, and Freund’s incomplete adjuvant were supplied by Sigma-Aldrich (St. Louis, MO, USA). Carboxymethoxyamine hemihydrochloride (CMO) was from Tokyo Chemical Industry (Tokyo, Japan). The monoclonal antibody isotyping kit was acquired from Wuhan Sanying Biotechnology Co., Ltd. (Wuhan, China). All other reagents were of analytical grade or higher without further purification.

The equipment includes a microplate reader (Shanghai Flash Spectrum Biotechnology Co., Ltd., Shanghai, China), a refrigerated centrifuge with high-speed (Sigma Laborzentrifugen GmbH, Osterode am Harz, Germany), an RH Digital KT/C magnetic stirrer (IKA-Werke GmbH & Co., KG, Staufen, Germany), a vacuum drying oven and electric blast drying oven (Shanghai Yiheng Technology Co., Ltd., Shanghai, China), an XYZ-3000 gold spraying and membrane-spotting instrument, as well as a CM 4000 cutter, (Bio-Dot, Inc., Irvine, CA, USA), HGS802 shell press (Hangzhou Fenghang Technology Co., Ltd., Hangzhou, China), and CSY-JAA colloidal gold analyzer (Shenzhen Finese Instruments Manufacturing Co., Ltd., Shenzhen, China).

### 2.2. Design and Synthesis of the Hapten

As a small-molecule toxin, ZEN requires derivatization to introduce a reactive functional group for covalent conjugation to a carrier protein during immunogen synthesis [24]. Carboxymethoxyamine (CMO) was used to convert ZAN into oxime hapten ZAN-O, which bears a terminal carboxyl group for protein coupling. Leveraging the structural homology among ZAN, ZEN, and their metabolites (α-ZEL, β-ZEL, and ZAL), a structural preservation strategy was adopted to retain the core scaffold and key functional moieties to maximize the cross-reactivity of the produced antibody with these analogs. This approach yielded high-affinity antibodies with broad specificity, which significantly enhance the detection sensitivity of ZEN and its derivatives in complex matrices.

**Figure 1:**
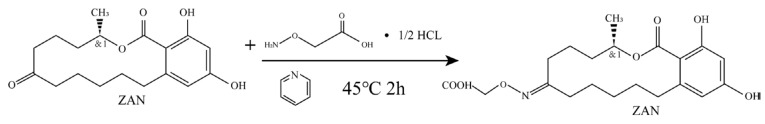
The preparation of ZAN-O immunogen.

Following Hu et al. [25], 2 mg of ZAN was dissolved in 2 mL of anhydrous pyridine, and 4 mg of CMO was added. The mixture was rotary evaporated at 45 °C for 2 h, and the residue was washed twice with methanol. After drying, the crude product was dissolved in 1 mL of double-distilled water, adjusted to pH 8.0, and extracted three times with equal volumes of toluene. The aqueous phase was further extracted three times with ethyl acetate, and the pooled organic extracts were evaporated to dryness to collect purified hapten ZAN-O (Figure 1).

For immunogen synthesis, purified ZAN-O hapten (dissolved in 0.5 mL DMF) was activated with 2 mg of EDC and 1.2 mg of NHS under stirring at room temperature for 2 h (Solution A). Then, 5 mg of bovine serum albumin (BSA) was dissolved in 0.5 mL of 0.3% NaHCO_3_ (Solution B). Under magnetic stirring, Solution A was added dropwise into Solution B and incubated for 2 h followed by dialyzing in PBS for 72 h to harvest the ZAN-O-BSA immunogen. The same amounts of ZAN, EDC, and NHS were reacted with an equal mass of OVA, which was dissolved in 0.3% NaHCO_3_ and stored overnight prior to use. Then, the prepared ZAN-O-OVA was used as the coating antigen for ELISA following the same procedure.

In contrast to most previously reported ZEN immunogens that directly modify ZEN or incorporate bulky spacer arms, this study derivatized ZAN through a CMO-oxime reaction to yield ZAN-O while maintaining the resorcylic acid lactone scaffold shared by ZEN and its major metabolites. Prioritizing the preservation of this conserved structural framework, the hapten was deliberately designed to favor broad-spectrum antibody recognition rather than narrow single-analyte specificity.

### 2.3. Preparation of Monoclonal Antibody

Female BALB/c mice (6–8 weeks old) were selected for immunization to generate the mAbs, according to the literature [26,27]. Four subcutaneous injections were conducted at three-week intervals. For the primary immunization, the prepared antigen was emulsified 1:1 (*v*/*v*) with Freund’s complete adjuvant (FCA) and injected subcutaneously into the dorsal neck and flank regions. Three booster injections were given under same conditions using the antigen emulsified with Freund’s incomplete adjuvant (FIA). Ten days after the final booster, blood was collected via tail vein puncture and centrifuged to obtain serum, which was diluted 1:10 (*v*/*v*) with PBS and used to determine the polyclonal antibody titers by indirect ELISA.

The mouse exhibiting the highest serum antibody was selected for hyperimmunization, and its splenocytes were harvested for fusion with SP2/0 myeloma cells using polyethylene glycol, as previously described [28]. The fused hybridoma cells were seeded into 96-well plates containing HAT selection medium and incubated for 7–10 days. Positive ZEN-specific hybridomas were identified by screening the culture supernatants via ELISA and were subsequently isolated by single-cell cloning using the limiting dilution method. The stable monoclonal antibody-producing cell lines were expanded in culture flasks and then intraperitoneally injected into BALB/c mice pretreated with paraffin one month earlier to induce ascites, from which the monoclonal antibodies were purified.

### 2.4. Preparation of Colloidal Gold and Gold-Labeled Antibody

Colloidal gold nanoparticles (AuNPs) were synthesized through the trisodium citrate reduction method [29,30]. The chloroauric acid (HAuCl_4_) aqueous solution was heated to boiling, after which trisodium citrate was quickly introduced under continuous stirring to act as both the reducing and stabilizing agent. The solution gradually turned from pale yellow to wine red, confirming successful AuNP formation. Once the color stabilized, the suspension was cooled to an ambient temperature and diluted to a desired volume with deionized water, and the final colloidal gold was stored at 4 °C for future use.

ZEN monoclonal antibody (ZEN mAb)–AuNP bioconjugates were prepared as follows. The colloidal gold solution (1 mL) was first adjusted to pH 8.0 with 0.2 M K_2_CO_3_ to achieve optimal conjugation conditions. Then, the ZEN mAb was added and incubated at room temperature for 30 min to form the mAb-AuNP bioprobe. To reduce nonspecific binding, 100 μL of 10% bovine serum albumin (BSA) was added to block the unbound active sites on the AuNPs. After centrifugation at 12,000 r/min for 20 min at 4 °C, the supernatant was discarded and the resulting pellet was gently resuspended in 100 μL of HB buffer to yield a 10× concentrated colloidal gold–ZEN mAb bioconjugate, which was stored at 4 °C until use.

### 2.5. Fabrication and Detection Mechanism of Colloidal Gold Immunochromatographic Assay

The colloidal gold immunochromatographic assay (CG-ICA) is a rapid analytical technique that integrates antigen–antibody specificity with colloidal gold-based colorimetric visualization and lateral flow chromatography. The test strip consists of four sequential components: a sample pad, conjugate pad, nitrocellulose (NC) membrane, and absorbent pad, all laminated onto a PVC backing. With an overlap of approximately 2 mm. On the NC membrane, the test line (T-line) features a ZEN-O-BSA conjugate for target capture, and the control line (C-line) contains Staphylococcal Protein A (SPA) to ensure the assay’s validity. After assembly, the strips are cut into 3 mm-width segments, dried under controlled conditions, sealed, and stored until use.

**Figure 2:**
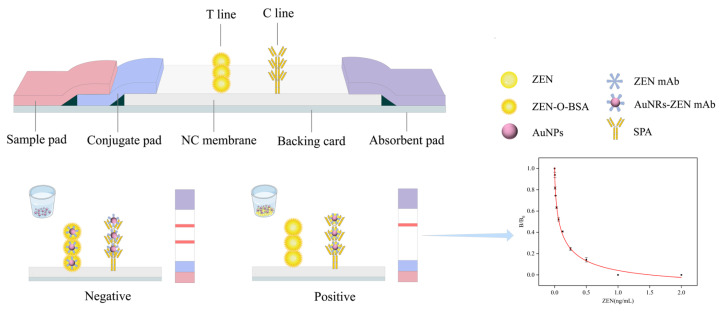
Schematic of the CG-ICA-based lateral flow assay for ZEN detection.

As depicted in Figure 2, the CG-ICA developed in this study employs a competitive immunoassay format. Briefly, 100 μL of the sample extract was added into a reaction vessel containing 1 μL of gold-labeled antibody and incubated for 5 min to ensure sufficient antigen–antibody interactions. Then, the test strip was inserted into the vessel to allow for the mixture to migrate along the test strip by capillary action. During migration, free ZEN in the sample competes with the immobilized ZEN-O-BSA on the T-line by occupying the binding sites of the gold-labeled antibody. At low or undetectable levels of free ZEN, the gold conjugates bind to the T-line, producing a distinct red band. As the ZEN concentration increases, fewer conjugates bind, resulting in a weaker or absent color signal. Notably, the C-line must consistently generate a visible signal regardless of ZEN levels; the absence of the C-line denotes an invalid assay.

### 2.6. Optimization of Detection Conditions

To improve the sensitivity and stability of the CG-ICA for ZEN detection, several key parameters were systematically optimized. The evaluation was based on T-line color intensity and the inhibition rate at ZEN concentrations of 2 ng/mL and 0.5 ng/mL, respectively. The parameters optimized included the pH of the reaction buffer, the concentration of the coating antigen, the dosage of mAb for gold labeling, the incubation time, the concentration factor of AuNPs for conjugation, and the concentration of SPA immobilized on the C-line. The signal intensities of the T-line and C-line were quantitatively measured using a Shenfen CSY-JAA colloidal gold analyzer, and the inhibition rate was calculated through the following formula:

(1) $$\begin{array}{c} \mathrm{Inhibition}\frac{rate}{\%}=\frac{B_{0}-B_{1}}{B_{0}}\times 100 \end{array}$$

where B_0_ represents the T-line signal intensity of the negative sample and B_1_ represents the T-line signal intensity of the positive samples.

The pH was systematically varied to modulate the surface charge and the colloidal stability of the AuNPs, thereby maximizing antibody adsorption efficiency and facilitating strong signal intensity with minimal background noise. Accordingly, different volumes (2, 4, 6, 8, 12, and 14 μL) of 0.2 M K_2_CO_3_ solution were added into 1 mL of colloidal gold solution to adjust the pH.

The concentration of the coating antigen immobilized on the T-line was varied (100, 50, 33, 25, and 20 μg/mL) to evaluate its effect on signal intensity and detection sensitivity. Antigen density directly influences binding efficiency in the competitive immunoassay; insufficient coating antigen produces a weak T-line signal, whereas excessive coating antigen can saturate binding sites, reducing sensitivity and complicating visual interpretation. The optimal coating concentration was therefore determined by correlating C-line and T-line coloration with inhibition rates using a strip reader.

The dosage of mAb used for gold labeling was varied (3, 4, 5, 6, and 7 μL per 1 mL of colloidal gold) to optimize sensitivity while maintaining colloidal stability. The optimal antibody volume was determined by balancing clear T-line visibility in negative controls with maximal inhibition rates in positive samples. Specifically, insufficient antibodies results in weak T-line signals and low sensitivity, while excessive antibodies can induce AuNP aggregation or nonspecific binding.

The incubation time between the sample and gold-labeled antibody was optimized at 2, 4, 6, 8, and 10 min to evaluate its effect on signal intensity and assay reproducibility. Reaction time is critical, too short an incubation compromises antigen–antibody binding efficiency and sensitivity, while excessively long incubation promotes nonspecific interactions or gold–antibody aggregation, both of which can reduce the background signal.

The concentration of AuNPs used for conjugation is also a critical influencing factor. Before conjugation, a 10× concentrated AuNP solution was diluted to 2×, 3×, 4×, and 5× to evaluate its effect on signal enhancement. Its concentration significantly influences signal intensity, and the optimal dilution ensures uniform migration along the test strip and clear visualization.

The concentration of SPA used for the C-line was varied at 0.2, 0.4, 0.6, and 0.8 mg/mL to ensure consistent C-line signal intensity and assay reliability. Insufficient SPA may lead to weak C-line intensity, risking failure, or ambiguous results, while excessive SPA can increase background noise and nonspecific adsorption. The optimal concentration was therefore selected to maintain clear C-line visibility while minimizing background interference, ensuring reliable assay performance.

### 2.7. Evaluation of Assay Performance

The sensitivity of the CG-ICA for ZEN detection was evaluated using a series of standard solutions in PBS buffer, comprising ten two-fold serial dilutions from 2.0 to 0.0039 ng/mL, with PBS alone as the negative control. The test strips were incubated with each standard solution, and the T-line intensity was assessed both by visually and quantitatively using a strip reader. For quantitative detection, a calibration curve was constructed by plotting the ZEN concentration (*x*-axis) against the signal ratio (B/B_0_, *y*-axis), where B represents the T-line signal intensity at each concentration and B_0_ corresponds to the baseline signal from the negative control. Based on the curve, the cut-off value and the half-inhibitory concentration (IC_50_) were calculated, defining the limit of detection and dynamic range for quantitative ZEN measurement.

The limit of detection (LOD), which was determined using 20 replicate measurements of negative samples, was confirmed by HPLC. The mean blank signal ($\bar{x}$, B_0_) and its standard deviation (SD) were calculated, and the LOD signal threshold was defined as $\bar{x}$-2SD (B). The minimum detectable *ZEN* concentration was determined by interpolating the normalized signal (B/B_0_) on the calibration curve. Then, the quantitative range was defined by the *ZEN* concentrations corresponding to normalized B/B_0_ between 0.2 and 0.8 on the standard curve. To assess the specificities of the *ZEN* mAb in the CG-ICA, the cross-reactivity (*CR*%) was determined against four common mycotoxins- aflatoxins B_1_ (AFB_1_), deoxynivalenol (DON), T-2 toxin (T-2), and ochratoxin A (OTA), and four structural analogs of ZEN include α-zearalenol (α-ZEL), β-zearalenol (β-ZEL), zearalanone (ZAN), and zearalol (ZAL), with each test at 100 ng/mL. The CR% was calculated following Wang et al. [31] using the following equation:

(2) $$\begin{array}{c} CR=\frac{IC_{50}\;of\;ZEN}{IC_{50}\;of\;other\;compounds}\times 100\% \end{array}$$

The precision was evaluated by evaluating both intra-batch and inter-batch reproducibility. IC_50_ values were measured for three independent batches, with each measurement repeated daily over a ten-day period. The intra- and inter-batch standard deviations (SDs) were calculated according to the *S*r formula, and the coefficient of variation (*CV*) was determined accordingly. These metrics provide a quantitative evaluation of the precision of the test strips. The formulas used for the calculations are as follows:

(3) $$S_{r}=\sqrt{\frac{1}{n-1}\sum_{i=1}^{n}\;(x_{i}-\bar{x}{)}^{2}}$$

where *n* represents the sample size, $\sum_{i=1}^{n}$ represents the summation from the first to the *n*-th data point, $x_{i}$ represents the value of the *i*-th data point, and $\bar{x}$ represents the sample mean.

(4) $$\begin{array}{c} CV=\frac{s}{\bar{x}}\times 100\% \end{array}$$

where *s* represents the sample standard deviation and $\bar{x}$ represents the sample mean.

### 2.8. Pretreatment of Maize Sample

Negative maize samples, confirmed by HPLC, were ground and divided into negative and positive groups, six independent replicates of 5 g. Positive samples were spiked with ZEN standard solution to a final concentration of 60 ng/kg. Each sample was extracted with 5 mL of a methanol–water mixture (*v*/*v*, 20%, 40%, 60%, 80%, and 100%) and agitated for 15 min to ensure thorough mixing. The extracts were then centrifuged at 6000 rpm for 5 min, and the supernatants were collected. Aliquots of each supernatant were serially diluted in PBS at ratios of 1:100, 1:200, 1:400, 1:800, and 1:1600, and the corresponding B/B_0_ values were plotted against the standard curve. The dilution factor corresponding to the IC_50_ was used to determine ZEN concentration in each sample. Subsequently, the recovery rates were calculated; the dilution that yielded a recovery rate closest to 100% was selected as optimal.

Then, the prepared extractions were used for the spiking experiment. ZEN standard solutions were added to the optimal extraction solution to achieve the final concentrations of 5, 10, and 20 μg/kg, respectively. The mixtures were shaken for 15 min to ensure complete homogenization, followed by centrifugation at 6000 rpm for 5 min to remove the precipitate. The resulting supernatants were diluted in PBS and analyzed. Spiked recovery rates were calculated based on ZEN concentrations determined from the calibration curve, confirming the accuracy and reproducibility of the method.

## 3. Results

### 3.1. Characterization of the Hapten

**Figure 3:**
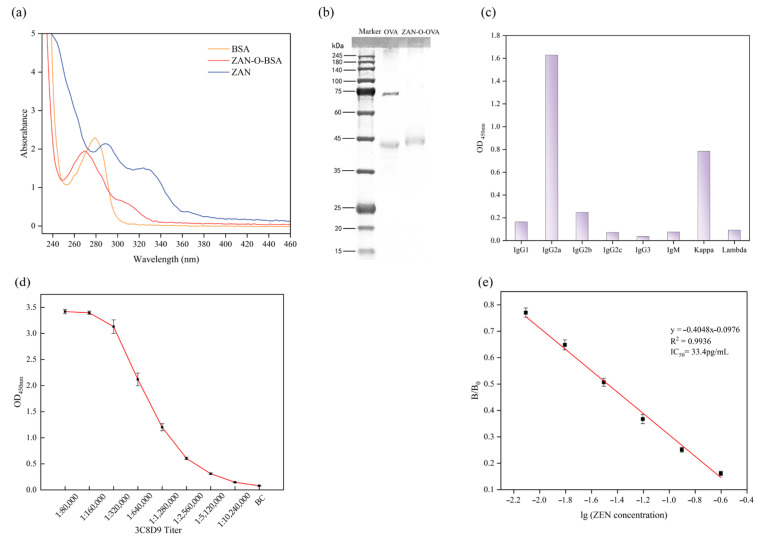
(**a**) UV-Vis spectral characterization of the ZAN artificial antigen; (**b**) SDS-PAGE analysis of the conjugation of ZAN-O-OVA; (**c**) isotype identification of the 3C8D9 monoclonal antibody; (**d**) 3C8D9 titer evaluation; (**e**) calibration curve for ZEN detection by the 3C8D9 monoclonal antibody.

To verify the successful synthesis of the hapten, ultraviolet–visible (UV–Vis) spectra of ZAN, BSA, and ZAN-O-BSA were recorded, as shown in Figure 3a. ZAN exhibited two well-defined absorption bands between 280 and 360 nm, with a pronounced maximum at approximately 285 nm. BSA displayed a characteristic band centered at 280 nm. The UV–Vis spectrum of the ZAN-O-BSA conjugate retains features of both ZAN and BSA, and, notably, showed a slight blue shift in the absorption maximum compared with free ZAN, consistent with perturbation of the π-conjugated system upon amide-bond formation and with the more polar protein microenvironment provided by BSA. This shift demonstrates that the ZAN is covalently attached to BSA, yielding a stable hapten–protein conjugate suitable for highly sensitive immunoassays. The artificial antigen ZAN-O-OVA was analyzed by SDS-PAGE (Figure 3b). The ZAN-O-OVA conjugate appeared as a broader and upshifted band, indicating increased molecular weight and reduced electrophoretic mobility, demonstrating the successful conjugation of ZAN-O to OVA.

### 3.2. Characterization of the Monoclonal Antibody

Upon immunization of mice with the synthesized antigen, a hybridoma clone, 3C8D9, was obtained, which stably secretes a monoclonal antibody with high sensitivity and specificity. The isotype’s titer, sensitivity, and antigen specificity were then determined.

Isotyping of the 3C8D9 monoclonal antibody was identified using a commercial monoclonal antibody isotyping kit to ascertain its subclass and light chain composition. As shown in Figure 3c, the antibody secreted from 3C8D9 was confirmed to be an IgG2a subclass with a κ light chain. This isotype combination is renowned for its optimal balance of serum stability, effector function activation, and is compatible with commonly used detection kits.

The titer of the antibody was determined by indirect ELISA using serial dilution of the purified ascites fluid; the endpoint was taken as the highest dilution. The results are shown in Figure 3d. The hybridoma clone 3C8D9 gave an endpoint titer of 1.28 × 10^6^, confirming both high antibody yield and strong antigen–antibody binding.

The sensitivity of the monoclonal antibody was quantified by indirect competitive ELISA under the following conditions: 15 min primary-antibody incubation, 30 min secondary-antibody incubation, and 10 min chromogenic development. The absorbance values were recorded using a microplate reader. Figure 3e presented the calibration curve for clone 3C8D9, following the equation y = −0.4048x − 0.0976 (R^2^ = 0.9936) and yielding an IC_50_ of 33.4 pg/mL, demonstrating exceptional sensitivity.

Specificity was evaluated by measuring cross-reactivity (CR) towards ZEN, its metabolites, and other common mycotoxins with the same assay by indirect competitive ELISA. The IC_50_ values for each analyte were measured, and CR was calculated, as given in Table 1. Notably, CR values > 100% for ZAN and ZAL are consistent with the fact that the monoclonal antibody was raised against the ZAN-O hapten and therefore recognizes these metabolites even more strongly than ZEN.

Clone 3C8D9 exhibited high cross-reactivity with the ZEN metabolites ZAL and ZAN (158.75% and 103.6%, respectively) and moderate CR toward α-ZEL (48.37%) and β-ZEL (24.91%), whereas negligible CR (<0.05%) was observed with the common mycotoxins AFB_1_, DON, T-2, and OTA. Compared with most reported anti-ZEN antibodies that predominantly recognize ZEN with minimal cross-reactivity, the monoclonal antibody 3C8D9 generated in this work shows strong affinity for ZAN and ZAL and moderate affinity for α-ZEL and β-ZEL. These results demonstrate that the ZAN-O based hapten design effectively broadens the molecular recognition spectrum, which is particularly valuable for food matrices where ZEN and its metabolites frequently coexist or interconvert. These results revealed that the 3C8D9 monoclonal antibody combines high specificity for ZEN with the effective recognition of its major metabolites, making the antibody ideal for comprehensive mycotoxin surveillance.

Taking together, the high titer, appropriate subclass, superior sensitivity, and outstanding specificity demonstrate that it is readily deployable in sensitive quantitative immunoassays for trace-level ZEN analysis in complex matrices.

### 3.3. Optimization of Colloidal Gold-Based Immunochromatographic Assay Parameters

To maximize assay performance, key parameters that influence the sensitivity of the CG-ICA were systematically evaluated. Optimization was guided by T-line intensity and the inhibition rate at ZEN concentrations of 2.0 and 0.5 ng/mL. The individual contributions of each parameter are detailed below to justify the final configuration.

**Figure 4:**
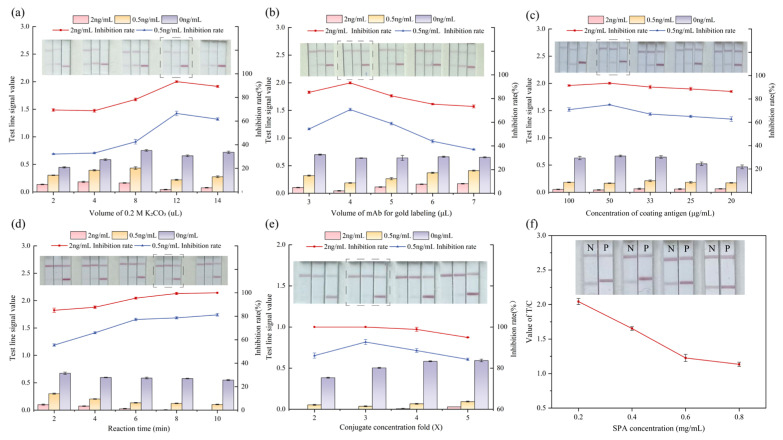
Parameter optimization of CG-ICA for ZEN detection based on signal intensity and inhibition rates at 2.0 and 0.5 ng/mL: (**a**) optimal experiment of pH by adding 0.2 M K_2_CO_3_; (**b**) dosage of mAb for gold labeling; (**c**) concentration of coating antigen ZAN-O-BSA on the T-line; (**d**) incubation time; (**e**) conjugate concentration fold; (**f**) concentration of SPA used for the C-line.

The pH of the gold labeling reaction system governs the electrostatic interaction between the antibody and AuNP surface and therefore dictates the adsorption efficiency. Because the as-prepared colloidal gold is weakly acidic, K_2_CO_3_ was added incrementally to raise the pH gradually, creating a more favorable environment for antibody conjugation. As shown in Figure 4a, the addition of 12 μL 0.2 M K_2_CO_3_ produced the most favorable pH, yielding the maximal probe stability and highest T-line inhibition at both ZEN concentrations of 2.0 and 0.5 ng/mL. This condition was adopted for all subsequent experiments.

The amount of mAb immobilized on AuNPs is another important determinant of assay performance. After screening, 4 μg/mL of mAb incubated with 1 mL colloidal gold was identified as optimal; the conjugate was concentrated three-fold before use. As shown in Figure 4b, the optimal working volume for antibody addition was determined to be 4 μL. This loading preserved immunoreactivity while avoiding antibody oversaturation, which increased nonspecific binding and compromised the antigen–antibody interaction. Under these conditions, the T-line was uniform and intense, permitting reliable detection of ZEN at the 0.5 ng/mL level.

The amount of ZAN-O-BSA immobilized on the T-line (20–100 μg/mL) was found to govern both signal visibility and assay sensitivity. As shown in Figure 4c, at high coating concentrations (e.g., 100 μg/mL), the strip produced an intense T-line in negative samples. However, excessive coating antigen resulted in relatively low inhibition rates even at elevated ZEN levels (2 ng/mL). Excess antigen presumably saturated the binding sites on the membrane surface, hampering displacement of the gold–antibody conjugate and reducing sensitivity. In contrast, lower concentrations (e.g., 20 or 25 μg/mL) produced faint lines that were difficult to read visually. Among the tested conditions, 50 μg/mL of ZAN-O-BSA yielded the best compromise, producing a well-defined T-line under negative conditions and demonstrating the highest inhibition at both 2.0 and 0.5 ng/mL of ZEN. This value was adopted for all subsequent work.

Allowing the sample and gold-labeled antibody to interact before migration strongly influenced the competition efficiency. As shown in Figure 4d, as the incubation time was prolonged, the inhibition rate gradually increased and the T-line intensity decreased, indicating progressive displacement. After incubation for 8 min, the inhibition rate reached its maximum, indicating that the reaction reached equilibrium and the competitive reaction was the most efficient. Longer times did not improve inhibition, and thus, 8 min was selected as the standard incubation period.

Probe density was adjusted by concentrating the gold–antibody pool. As shown in Figure 4e, a three-fold concentration of the gold-labeled antibody solution gave the sharpest increase in inhibition while preserving a uniform T-line in negative strips. This condition ensures adequate probe availability for effective antigen–antibody interactions without inducing steric hindrance or nanoparticle aggregation. Higher concentrations (four-fold or five-fold) introduced irregular staining and a slight drop in inhibition, most likely because nanoparticle overcrowding hindered lateral flow and promoted nonspecific trapping. Therefore, a three-fold concentration was used to balance sensitivity, line definition, and reproducibility.

The SPA immobilized on the C-line acts as a built-in flow and conjugate control. As shown in Figure 4f, SPA concentrations ranging from 0.2 to 0.8 mg/mL were compared for line sharpness and T/C contrast. At 0.8 mg/mL, the C-line was slightly broader and more intensely colored than the T-line, providing optimal visual reference without excess reagent. Higher loadings did not intensify the signal and were therefore unnecessary.

The sequential optimization yielded the following standard conditions: 12 μL 0.2 M K_2_CO_3_, 50 μg/mL coating antigen on the T-line, 4 μL of mAb per 1 mL of colloidal gold, 8 min of sample/conjugate incubation, three-fold concentration of the conjugate before application, and 0.8 mg/mL of SPA on the C-line. These settings collectively improved the detection limit, inter-strip precision, and visual discriminability, providing a robust platform for on-site screening of ZEN.

### 3.4. Analytical Performance of the CG-ICA for ZEN Detection

**Figure 5:**
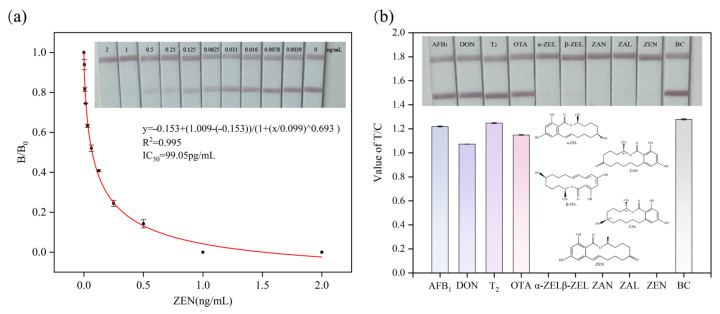
(**a**) Standard curve for ZEN detection by CG-ICA; (**b**) specificity evaluation of CG-ICA.

Under the optimized conditions, a series of ZEN standard solutions (2.0, 1.0, 0.5, 0.25, 0.125, 0.063, 0.03126, 0.0156, 0.0078, and 0.0039 ng/mL) were prepared in PBS and analyzed in parallel with a blank control. Each standard solution was incubated with the gold-labeled antibody for 8 min, then applied for the immunochromatographic assay. A calibration curve was constructed by plotting the T-line responses against ZEN concentrations, and the data fitting was performed using a four-parameter logistic model. The standard curve exhibited an excellent correlation coefficient (R^2^ = 0.995), with an IC_50_ value of 99.06 pg/mL. The visual detection limit (cutoff value) was determined to be 1 ng/mL, highlighting the high sensitivity of the developed CG-ICA (Figure 5a).

The assay’s sensitivity was evaluated through 20 repeated analyses of ZEN-free maize samples. The results are shown in Table 2. According to the above calibration curve, the limit of detection (LOD) was calculated to be 11.79 pg/mL, and ZEN concentrations corresponding to B/B_0_ values of 0.2 and 0.8 were 13.40 pg/mL and 732.48 pg/mL, establishing a quantitative working range of 13.40–732.48 pg/mL.

Table 3 summarizes the performance of recently reported ZEN assays. The present CG-ICA offers one of the lowest LODs and broad linear range, while retaining operational simplicity, rapidity, and minimal instrumentation requirements. These features make the strip ideally suited for the on-site screening of maize and other cereals without specialized laboratories. In comparison with many recently reported highly sensitive ZEN LFIAs that depend on complex nanomaterials such as Au@PDA [32], nanozymes [33], AIE probes [34], or magnetic particles [35]) or require additional signal-amplification reagents, the proposed CG-ICA achieves a sensitivity of 11.79 pg/mL solely through systematic optimization of conventional colloidal gold antibody conjugates and strip parameters. These findings confirm that high analytical sensitivity can be achieved without resorting to complex nanomaterials or auxiliary amplification strategies, while fully retaining the inherent advantages of conventional CG-ICA, including straightforward operation, low cost, and robust applicability to on-site detection.

### 3.5. The Specificity and Stability

The cross-reactivity (CR) of the optimized CG-ICA was examined against both structurally related and unrelated mycotoxins. As shown in Figure 4b, when non-analogs (AFB_1_, DON, T-2, and OTA, 100 ng/mL) were tested, the T-line intensity was indistinguishable from that of the blank, corresponding to a CR < 0.05%, as shown in Table 4, confirming negligible interference.

For structural analogs (α-ZEL, β-ZEL, ZAN, and ZAL, 100 ng/mL), the assay exhibited varying degrees of CR with ZEN. Notable T-line inhibition was observed, suggesting partial cross-recognition by the mAb. Corresponding CRs were calculated and presented in Table 4. This can be attributed to the shared core structure, a phenolic ring, and lactone moiety, which facilitates recognition by the mAb via hydrophobic and hydrogen-bonding interactions. Because ZEN can convert into ZAN and ZAL during food processing, the antibody’s broad-spectrum reactivity provides a more comprehensive monitoring of ZEN and its metabolites and improves risk-assessment accuracy.

The methodological stability was evaluated by calculating both the intra- and inter-batch coefficients of variation (CVs). As summarized in Table 5, the intra-batch CVs for three independently prepared replicates remained below 15%, indicating high repeatability under uniform conditions. The inter-batch CVs also remained below 15%, confirming excellent reproducibility and repeatability across separate assay batches. These results demonstrate satisfactory repeatability and reproducibility for the routine quantification of ZEN.

### 3.6. Detection of ZEN in Maize Samples

To optimize sample pretreatment conditions, the extraction efficiency of ZEN from maize was evaluated using methanol–water mixtures at various concentrations, as shown in Table 6. At low methanol concentrations (20–40%), the recovery rates were below 20%, failing to meet the sensitivity requirements of the assay. The extraction efficiency improved markedly by increasing methanol content and reached a maximum at 80% methanol, with a recovery rate of 95.81%. Higher concentrations did not improve extraction but increased matrix interference; consequently, 80% methanol was selected as the optimal extraction solvent for ZEN.

Under the optimized extraction conditions, further evaluation was conducted to verify the analytical performance of the CG-ICA method through spiked recovery experiments. Negative maize samples were spiked with ZEN at concentrations of 5, 10, and 20 μg/kg, followed by extraction with 80% methanol and subsequent analysis using the established protocol. As depicted in Table 7, the recovery rates ranged from 85.36% to 98.86%, with coefficients of variation (CVs) consistently below 10%, demonstrating excellent method accuracy and precision. Importantly, even at the lowest fortification level of 5 μg/kg, the assay yielded a high recovery rate and minimal variability, underscoring its suitability for the detection of trace-level ZEN residues in maize samples.

Thus, the CG-ICA platform offers an instrument-free field-ready tool for ZEN monitoring in maize. Combining sub-nanogram sensitivity and operational simplicity, it ideally suited for large-scale application in food quality inspection workflows, providing an early warning capacity for regulatory compliance across cereal supply chains.

## 4. Conclusions

A colloidal gold immunochromatographic assay (CG-ICA) test strip was successfully developed for the rapid and sensitive detection of zearalenone (ZEN) in maize samples. After systematic optimization, the assay obtained high specificity and sensitivity, which achieved a broad linear range of 13.40–732.48 pg/mL and a low detection limit (LOD) of 11.79 pg/mL, facilitating effective screening of ZEN residues in complex food matrices. Notably, the test strip exhibited notable cross-reactivity with major ZEN metabolites, enhancing its applicability for comprehensive mycotoxin surveillance. The CG-ICA platform holds considerable promise for routine food safety monitoring and may serve as a field-ready tool for early warning and compliance verification across cereal supply chains.

## Figures and Tables

**Table 1:** Cross-reactivity of 3C8D9 monoclonal antibody.

Inhibitors	IC_50_ (pg/mL)	CR (%)
ZEN	33.40	100.00
ZAL	21.04	158.75
ZAN	32.24	103.60
α-ZEL	69.05	48.37
β-ZEL	134.09	24.91
OTA	>1 × 10^5^	<0.05
DON	>1 × 10^5^	<0.05
T-2	>1 × 10^5^	<0.05
AFB_1_	>1 × 10^5^	<0.05
BSA	>1 × 10^5^	<0.05
OVA	>1 × 10^5^	<0.05

**Table 2:** Limit of detection (LOD) determination for ZEN colloidal gold test strip.

	Test Line Intensity									
	0.50	0.46	0.37	0.37	0.37	0.36	0.34	0.38	0.36	0.34
	0.40	0.37	0.40	0.35	0.37	0.40	0.42	0.42	0.42	0.36
$\bar{x}$	0.3858									
SD	0.0400									
$\bar{x}$-2SD	0.3058									

**Table 3:** Comparative analytical performance of reported methods for ZEN detection.

Method	Linear Range (ng/mL)	Limit of Detection (LOD, ng/mL)	Reference
Molecularly Imprinted Electrochemical Sensor	1–500	0.34	[36]
Fluorescence Polarization Aptamer Sensor	0.01–100	0.004	[37]
Surface-Enhanced Raman Spectroscopy	5–400	3	[38]
Surface Plasmon Resonance	1–480	0.102	[39]
Enzyme-Linked Immunosorbent Assay (ELISA)	0.19–1.51	0.54	[40]
Quantum Dot Fluorescent Immunochromatography	0.264–23.55	0.125	[41]
SERS-based lateral flow immunosensor	0–1000	3.6	[42]
This Method	0.0134–0.7325	0.0118	This work

**Table 4:** Cross-reactivity rate of CG-ICA test strip.

Inhibitors	IC_50_ (pg/mL)	CR (%)
ZEN	99.05	100.00
ZAL	86.23	114.85
ZAN	95.98	103.15
α-ZEL	239.8	41.28
β-ZEL	840.4	11.78
OTA	>1 × 10^5^	<0.05
DON	>1 × 10^5^	<0.05
T-2	>1 × 10^5^	<0.05
AFB_1_	>1 × 10^5^	<0.05

**Table 5:** Precision analysis of CG-ICA test strip.

Batch	Intra-Batch Standard Deviation	CV (100%)	Inter-Batch Standard Deviation	CV (%)
1	13.42	13.81	8.08	8.22
2	9.92	10.93		
3	12.43	11.65		

**Table 6:** Evaluation of extraction solvent for ZEN recovery from maize samples.

Methanol Concentration (%)	20	40	60	80	100
ZEN Content (µg/kg)	5.26	10.21	40.85	57.49	54.34
Extraction Efficiency (%)	8.77	17.02	68.09	95.81	90.57

**Table 7:** Determination of ZEN-spiked maize samples by CG-ICA method.

Spiking Level (μg/kg)	Recovered Amount (μg/kg)	Recovery Rate (%)	CV (%)
5	4.27	85.36	6.98
10	8.73	87.30	8.34
20	19.77	98.86	7.33

## Data Availability

The data presented in this study are available in this article or upon reasonable request to the corresponding author (Mei Hu).

## Abbreviations

The following abbreviations are used in this manuscript:

CG-ICA	Colloidal gold immunochromatographic assay
OVA	Ovalbumin
BSA	Bovine albumin
AuNPs	Colloidal gold nanoparticles
